# Internal Medicine Point-of-Care Ultrasound Curriculum: Consensus Recommendations from the Canadian Internal Medicine Ultrasound (CIMUS) Group

**DOI:** 10.1007/s11606-017-4071-5

**Published:** 2017-05-11

**Authors:** Irene W. Y. Ma, Shane Arishenkoff, Jeffrey Wiseman, Janeve Desy, Jonathan Ailon, Leslie Martin, Mirek Otremba, Samantha Halman, Patrick Willemot, Marcus Blouw, On behalf of The Canadian Internal Medicine Ultrasound (CIMUS) Group*, Khalid Azzam, Marko Balan, Sharon E. Card, Tara Cessford, Barry Chan, Darrel Cotton, Colin R. Gebhardt, Neil E. Gibson, Catherine J. Gray, Rajender Hanmiah, Babar A. Haroon, Dev Jayaraman, Alexandre Lafleur, Ada Lam, Jed Lipes, Michael Mayette, Steven J. Montague, Hassan Mustafa, Leanne Reimche, Jennifer Ringrose, Stephanie Rodrigue, Mireille Sayegh, Jeffrey P. Schaefer, Steven Shadowitz, Stephanie Tom

**Affiliations:** 1 Department of Medicine, University of Calgary, Calgary, AB Canada; 2 W21C, University of Calgary, Calgary, AB Canada; 3 University of British Columbia, Vancouver, BC Canada; 4 McGill University, Montreal, QC, Canada; 5 University of Toronto, Toronto, ON Canada; 6 McMaster University, Hamilton, ON Canada; 7 University of Ottawa, Ottawa, ON Canada; 8 University of Manitoba, Winnipeg, MN Canada

**Keywords:** point-of-care ultrasound, internal medicine, curriculum

## Abstract

Bedside point-of-care ultrasound (POCUS) is increasingly used to assess medical patients. At present, no consensus exists for what POCUS curriculum is appropriate for internal medicine residency training programs. This document details the consensus-based recommendations by the Canadian Internal Medicine Ultrasound (CIMUS) group, comprising 39 members, representing 14 institutions across Canada. Guiding principles for selecting curricular content were determined a priori. Consensus was defined as agreement by at least 80% of the members on POCUS applications deemed appropriate for teaching and assessment of trainees in the core (internal medicine postgraduate years [PGY] 1–3) and expanded (general internal medicine PGY 4–5) training programs. We recommend four POCUS applications for the core PGY 1–3 curriculum (inferior vena cava, lung B lines, pleural effusion, and abdominal free fluid) and three ultrasound-guided procedures (central venous catheterization, thoracentesis, and paracentesis). For the expanded PGY 4–5 curriculum, we recommend an additional seven applications (internal jugular vein, lung consolidation, pneumothorax, knee effusion, gross left ventricular systolic function, pericardial effusion, and right ventricular strain) and four ultrasound-guided procedures (knee arthrocentesis, arterial line insertion, arterial blood gas sampling, and peripheral venous catheterization). These recommendations will provide a framework for training programs at a national level.

## INTRODUCTION

The use of point-of-care ultrasound (POCUS) has increased significantly over the last decade. This is likely the result of accumulating evidence demonstrating that effective POCUS skills can be acquired with minimal training^1^^–^ 3 and that POCUS may improve diagnostic performance when used with the traditional physical examination,^4^^–^ 6 especially in situations where patient characteristics limit the accuracy of the physical examination.^7^^,^ 8

POCUS has significant utility in assessing patients seen by internists. For the assessment of dyspneic patients, POCUS has demonstrated higher accuracy than the traditional workup.^6^^,^ 9 For example, the use of POCUS for assessment of lung B lines in heart failure patients on discharge can predict readmission rates at 6 months.^10^ Second, POCUS guidance of some bedside procedures reduces errors and complications.^11^^–^ 13 Lastly, POCUS use in internal medicine may result in reduced expenditures.^14^

The performance of POCUS is highly operator-dependent, and appropriate competency-based training is necessary prior to its use.^15^^,^ 16 Despite the purported clinical benefits of POCUS, however, there is currently no clear agreement as to what an internal medicine POCUS curriculum should be.^17^^,^ 18 To address this gap,^19^ this document outlines a set of consensus-based recommendations for a Canadian internal medicine POCUS curriculum, accounting for the existing limitations in available trained faculty, administrative structures, and resources within a Canadian context.

## METHODS

The Canadian Internal Medicine Ultrasound (CIMUS) group comprises leadership representatives from a number of internal medicine residency programs across Canada. Support for this group’s work was obtained from the Canadian Society of Internal Medicine (CSIM) Council and Education Committee in October 2015. In June 2016, program directors from each of 17 Canadian internal medicine residency training programs (postgraduate years [PGY] 1–3) and 16 general internal medicine residency training programs (PGY 4–5), as well as 17 internal medicine division chiefs across Canada, were invited to identify leaders in their respective programs and/or divisions as having specialized POCUS skills, educational expertise, and/or leadership roles within their institution for advancing POCUS use and education within internal medicine.^20^^,^ 21 Each identified lead was then invited to participate in a 4-h consensus meeting, using a modified nominal group technique (NGT),^22^ held during the CSIM Annual Meeting in Montréal, QC, on October 29, 2016. Those unable to attend the meeting in person participated via teleconference. The Royal College of Physicians and Surgeons of Canada (RCPSC) is Canada’s national accreditation body for residency programs. As such, one representative each from the RCPSC specialty committees in internal medicine and general internal medicine also participated in this meeting.

At the meeting, preliminary learner needs assessment data from five Canadian internal medicine training programs were presented. Participants then discussed and agreed upon four overarching principles upon which curricular items would be selected:

1. Applications should be selected based on clinical and/or educational needs.
2. Applications should be educationally feasible (i.e. both the cognitive and technical components of the application can be reasonably taught and learned in a competency-based manner, considering existing resource limitations).
3. Content should have clinical and/or educational evidence to support its use.
4. In the adoption of its use, any unintended clinical consequences should pose minimal risks to patients and/or it should include methods that can be implemented to minimize risks (e.g. program policies).

To this end, we aimed to achieve the *minimum* number of topics that we felt could feasibly be introduced, given existing limitations in equipment resources, trainee time, and expert faculty time. The process of voting (described below) was then discussed with the expert group. We determined a priori to conduct no more than three rounds of voting.^22^

The meeting was facilitated by two POCUS experts (IM, SA), both of whom have completed a 1-year dedicated POCUS fellowship. At the start of the meeting, a list of candidate POCUS applications (25 applications and 10 ultrasound-guided procedures) was presented based on commonly accepted POCUS applications^23^^–^ 26 and Canadian internal medicine procedural competency training requirements.^27^^,^ 28 Paper copies of key articles were also provided at the meeting.^23^^–^ 28 We did not conduct a round-robin discussion for item generation, given the existence of commonly accepted applications. Our participant group size (*N* = 39) was substantially larger than group sizes typically used in NGT studies (*N* = 5–12).^22^ To optimize participant engagement in the discussion of each of the 35 curricular items, we divided participants into five subgroups rather than having one large group discussion.

Following the small group discussions, a preliminary large group discussion was held on individual curriculum applications, led by the same facilitators (IM, SA). Participants then voted anonymously on each item as to whether it should be included in or excluded from a core internal medicine POCUS curriculum (postgraduate years [PGY] 1–3) or expanded general internal medicine curriculum (PGY 4–5). All participants voted using an anonymous paper-based approach (or via e-mail for the teleconference participants). We defined consensus as agreement by at least 80% of the members. This 80% threshold is in keeping with guideline recommendations.^29^

All applications not reaching consensus were put forward for consideration by voting in round 2. Only quantitative results (percentage agreement) for applications that did not reach consensus were fed back to the panel. For each of these applications, if more than 50% of participants indicated interest in readdressing it, the application was voted upon again in round 2. The second round was conducted in an open, unblinded fashion (i.e. not anonymous) for convenience reasons due to time limitations (a maximum of 4 h was allotted for the meeting). Items with 80% or greater agreement were considered to have reached consensus. A final round was then conducted using an online survey in a blinded fashion approximately 2 weeks after the meeting in order to minimize the potential impact of dominating members of the group on the unblinded second-round vote.^22^ The same experts were invited to participate in all rounds.

## RESULTS

A total of 47 individuals were identified by 14 of the 17 (82%) Canadian academic institutions as meeting POCUS education leadership criteria. Of these, 39 (83%) individuals participated in the meeting: 31 in person and eight via teleconferencing. Baseline demographics of the 39 individuals are described in Table 1.

**Table 1:** Demographics of the 39 Members of the Canadian Internal Medicine Ultrasound Group

Demographic	Number (%)*
Academic institution	
University of British Columbia	2 (5)
University of Calgary	6 (15)
University of Alberta	4 (10)
University of Saskatchewan	2 (5)
University of Manitoba	1 (3)
Northern Ontario School of Medicine	0
Western University	1 (3)
McMaster University	4 (10)
University of Toronto	4 (10)
Queen’s University	2 (5)
University of Ottawa	4 (10)
McGill University	4 (10)
Université de Montréal	0
Université de Sherbrooke	1 (3)
Université Laval	2 (5)
Dalhousie University	1 (3)
Memorial University of Newfoundland	0
Province	
British Columbia	2 (5)
Alberta	10 (26)
Saskatchewan	2 (5)
Manitoba	1 (3)
Ontario	15 (38)
Québec	7 (18)
Nova Scotia	1 (3)
Newfoundland and Labrador	0
Gender	
Male	25 (64)
Female	14 (36)
Subspecialty^†^	
General internal medicine	34 (87)
Critical care medicine	4 (10)
Nephrology	2 (5)
Cardiology	1 (3)
Rheumatology	1 (3)
Years of practice ***using*** ultrasound^‡^	
1–2 years	6 (18)
3–5 years	13 (39)
6–10 years	8 (24)
11 or more	3 (9)
Years of experience ***teaching*** ultrasound^‡^	
1–2 years	12 (36)
3–5 years	8 (24)
6–10 years	5 (15)
11 or more	0
Years of experience ***assessing*** ultrasound^‡^	
1–2 years	13 (39)
3–5 years	8 (24)
6–10 years	2 (6)
11 or more	0
Completed a 1-year (or more) dedicated ultrasound fellowship	8 (24)
Completed a fellowship where ultrasound was taught	7 (21)

*Not all individuals responded to all the questions^†^Individuals could choose more than one subspecialty^‡^Only 33 individuals responded to this portion of the survey

### Round 1

A total of 25 POCUS applications and ten procedures were considered (Table 2). Thirty-five of the 39 members (90%) voted in round 1, as not all individuals were able to participate in the meeting in its entirety. Consensus for inclusion was reached for four applications (inferior vena cava, B lines, pleural effusion, and abdominal free fluid) and three procedures (central venous catheterization, thoracentesis, and paracentesis) for the core internal medicine (PGY 1–3) curriculum (Table 2).

**Table 2:** Results of First Round of Consensus Meeting: Votes by Members (*n* = 35) on Each Application

Round 1 items	Include in core, no. (%)	Include in expanded, no. (%)	Should **NOT** include, no. (%)
Volume status			
Internal jugular vein (for jugular venous pressure assessment)	21 (60)	25 (71)	10 (29)
Inferior vena cava	30 (86)^†^	34 (97)^†^	1 (3)
Lung			
B lines	28 (80)^†^	34 (97)^†^	1 (3)
Pleural effusion	35 (100)^†^	35 (100)^†^	0 (0)
Consolidation	7 (20)	32 (91)^†^	3 (9)
Pneumothorax	10 (29)	31 (89)^†^	4 (11)
Abdomen			
Free fluid/ascites	35 (100)^†^	35 (100)^†^	0 (0)
Biliary pathology	0	4 (11)	31 (89)^†^
Bowel obstruction	0	3 (9)	32 (91)^†^
Renal/genitourinary			
Hydronephrosis	4 (11)	17 (49)	18 (51)
Bladder	17 (49)	24 (69)	11 (31)
Soft tissue/musculoskeletal			
Abscess*	3 (9)	13 (39)	21 (64)
Cobblestoning*	0	8 (24)	26 (76)
Knee effusion*	8 (24)	28 (82)^†^	6 (18)
Shoulder effusion*	2 (6)	9 (27)	24 (73)
Shoulder impingement*	0	2 (6)	32 (94)^†^
Synovitis*	0	2 (6)	31 (94)^†^
Cardiac			
Gross left ventricular systolic function	17 (49)	32 (91)^†^	3 (9)
Pericardial effusion	21 (60)	31 (89)^†^	3 (9)
Right ventricular strain	6 (17)	24 (69)	11 (31)
Valvular lesions	1 (3)	8 (23)	27 (77)
Vascular			
Abdominal aortic aneurysm	3 (9)	9 (26)	26 (74)
Deep vein thrombosis	1 (3)	10 (29)	25 (71)
Ocular			
Optic nerve diameter	0	4 (11)	31 (89)^†^
Pupillary reflex	0	2 (6)	33 (94)^†^
Procedure guidance			
Central venous catheterization	34 (97)^†^	35 (100)^†^	0
Thoracentesis	34 (97)^†^	35 (100)^†^	0
Paracentesis	34 (97)^†^	35 (100)^†^	0
Knee arthrocentesis	6 (17)	31 (89)^†^	4 (11)
Lumbar puncture*	4 (12)	21 (62)	13 (38)
Arterial line insertion	16 (46)	26 (74)	9 (26)
Arterial blood gas sampling	12 (34)	20 (57)	15 (43)
Peripheral venous catheterization	14 (40)	21 (60)	14 (40)
Assessment for intubation*	1 (3)	5 (15)	29 (85)^†^
Abscess drainage/aspiration	2 (6)	10 (29)	25 (71)

*Not all individuals voted for this item^†^Consensus achieved

For the expanded (PGY 4–5) curriculum, consensus for inclusion was reached for nine applications (the same four core PGY 1–3 applications plus lung consolidation, pneumothorax, knee effusion, gross left ventricular systolic function, and pericardial effusion) and four procedures (three core PGY 1–3 procedures plus knee arthrocentesis).

Six applications (biliary pathology, bowel obstruction, shoulder impingement, synovitis, optic nerve diameter, and pupillary reflex) and one procedure (POCUS assessment for intubation) reached consensus for exclusion from both the core PGY 1–3 and the expanded PGY 4–5 curricula.

For the remaining items, there was no consensus on either inclusion or exclusion with respect to the core PGY 1–3 curriculum. Of these applications, more than 50% of the group voted to readdress seven applications (internal jugular vein, pneumothorax, gross left ventricular systolic function, pericardial effusion, right ventricular strain, abdominal aortic aneurysm, and deep vein thrombosis) and five procedures (knee arthrocentesis, lumbar puncture, arterial line insertion, arterial blood gas sampling, and peripheral venous catheterization).

### Round 2

Thirty-four experts voted in round 2. No additions were made regarding the core PGY 1–3 applications after voting on these seven topics and five procedures in round 2. For the expanded PGY 4–5 curriculum, two additional applications reached consensus for inclusion—internal jugular venous height and right ventricular strain—resulting in a total of 11 topics for the expanded PGY 4–5 curriculum. In addition, three new procedures reached consensus for inclusion in the expanded PGY 4–5 curriculum: arterial line insertion, arterial blood gas sampling, and peripheral venous catheterization.

### Round 3

In the last round, 38 of 39 (95%) members participated via a blinded online survey approximately 2 weeks after the initial meeting. Consensus remained for all the final items from round 2, which included four applications and three procedures for the core PGY 1–3 curriculum and 11 applications and seven procedures for the expanded PGY 4–5 curriculum (Table 3).

**Table 3:** Results of Final Round of Consensus Meeting: Votes by Members (*n* = 38) on Items for Inclusion in the Core (PGY 1–3) and Expanded (PGY 4–5) Curricula

	Voted to include, no. (%)	Voted to exclude, no. (%)
Core PGY 1–3 Curriculum		
Volume status		
Inferior vena cava*	35 (95)	2 (5)
Lung		
B lines	36 (95)	2 (5)
Pleural effusion	38 (100)	0
Abdomen		
Free fluid/ascites	38 (100)	0
Procedure guidance		
Central venous catheterization	37 (97)	1 (3)
Thoracentesis	38 (100)	0
Paracentesis	38 (100)	0
Expanded PGY 4–5 Curriculum^†^		
Volume status		
Internal jugular vein*	32 (86)	5 (13)
Lung		
Consolidation	36 (95)	2 (5)
Pneumothorax	36 (95)	2 (5)
Soft tissue/musculoskeletal		
Knee effusion	33 (87)	5 (13)
Cardiac		
Gross left ventricular systolic function	38 (100)	0
Pericardial effusion	38 (100)	0
Right ventricular strain	33 (87)	5 (13)
Procedure guidance		
Knee arthrocentesis	32 (84)	6 (16)
Arterial line insertion	35 (92)	3 (8)
Arterial blood gas sampling	33 (87)	5 (13)
Peripheral venous catheterization	31 (82)	7 (18)

*PGY* postgraduate year

*Not all individuals voted for this item^†^All applications included in the core PGY 1–3 curriculum are also to be included in the expanded PGY 4–5 curriculum

## DISCUSSION

We recommend that four applications (inferior vena cava, lung B lines, pleural effusion, and abdominal free fluid) and three procedures (central venous catheterization, thoracentesis, and paracentesis) be included in the core Internal Medicine PGY 1–3 curriculum. For the expanded PGY 4–5 curriculum, we recommend that in addition to the core applications and procedures listed above, seven applications (internal jugular vein, lung consolidation, pneumothorax, knee effusion, gross left ventricular systolic function, pericardial effusion, and right ventricular strain), and four procedures (knee arthrocentesis, arterial line insertion, arterial blood gas sampling, and peripheral venous catheterization) be included.

A number of contextual features and limitations should be highlighted in the interpretation and application of our results. First, our group aimed to achieve the *minimum* number of topics that we felt could feasibly be introduced, given the existing limitations in resources, trainee time, and expert faculty within the Canadian internal medicine programs.^30^ These guidelines are not intended to dissuade programs from teaching additional applications. Second, these recommendations are expected to change over time. As programs gain comfort and expertise, and as additional evidence on POCUS becomes available, we anticipate that our current recommendations will need to be modified. With time, we anticipate that some of these applications will be taught in the undergraduate medical curriculum^31^ and may need only to be reviewed in the postgraduate curriculum. Third, our recommendations were determined solely by expert opinion-based consensus. We did not grade the strength of our recommendations or conduct a systematic review of all applications. However, collectively, we feel that our group has the necessary clinical and educational expertise and awareness of our current training limitations to make the above recommendations. Fourth, because of the large number of items considered and the number of experts in our group, we chose to ask the experts to indicate binary responses (should include vs. should not include) rather than ranking or rating items on Likert scales. Future studies could consider these alternative rating options. Fifth, because of the anonymous nature of the process, we were not able to identify which experts did or did not participate in the voting for each round, only that we had response rates of 90% in round 1, 87% in round 2, and 97% in round 3. Future studies should consider tracking the identities of each expert. Sixth, our report does not cover curriculum design or implementation issues.

### Future Directions

Having established these consensus-based curricula, the next steps in curriculum development will involve setting goals and objectives, designing educational strategies, implementing the curriculum, and evaluating the program.^19^ National scanning standards should also be defined in addition to the development of competency-based assessment procedures. As a group, we are committed to future work listed above. In November 2016, we submitted our curricula recommendations to the Royal College of Physicians and Surgeons of Canada for consideration for inclusion in the internal medicine and general internal medicine documentation. Lastly, for the Canadian programs, we recommend that a competency-based curriculum be in place for the above applications by the year 2020.

## CONCLUSIONS

As a pan-Canadian internal medicine expert-based group, the Canadian Internal Medicine Ultrasound (CIMUS) group has reached consensus on the POCUS applications for internal medicine postgraduate curriculum. We recommend that four POCUS applications and three procedures be included in the core PGY 1–3 curriculum, and 11 POCUS applications and seven procedures be included in the expanded PGY 4–5 curriculum.
